# Three-dimensional fine structure of calcified nodules in the common femoral artery based on low-vacuum scanning electron microscopy

**DOI:** 10.1093/ehjcr/ytae196

**Published:** 2024-04-15

**Authors:** Masanori Nishimura, Mitsuhiro Yano, Kensaku Nishihira, Atsuko Yokota, Yujiro Asada, Akira Sawaguchi

**Affiliations:** Department of Cardiovascular Surgery, Miyazaki Medical Association Hospital, Miyazaki, Japan; Department of Cardiovascular Surgery, Miyazaki Medical Association Hospital, Miyazaki, Japan; Department of Cardiology, Miyazaki Medical Association Hospital, 1173 Arita, Miyazaki 880-2102, Japan; Department of Cardiovascular Surgery, Miyazaki Medical Association Hospital, Miyazaki, Japan; Department of Diagnostic Pathology, Miyazaki Medical Association Hospital, Miyazaki, Japan; Department of Anatomy, Faculty of Medicine, University of Miyazaki, Miyazaki, Japan

A 75-year-old man with diabetes mellitus and hypertension complained of intermittent claudication in the right lower extremity; its ankle–brachial index was markedly decreased (0.45; normal range: 1.00–1.40). Angiography showed severe stenosis with a contrast defect in the right common femoral artery (CFA) (arrows, *Panel A*; Supplementary material online, *Video S1*). The lesion was highly calcified on computed tomography. We decided to perform endarterectomy for the calcified lesion in the right CFA.

**Figure:**
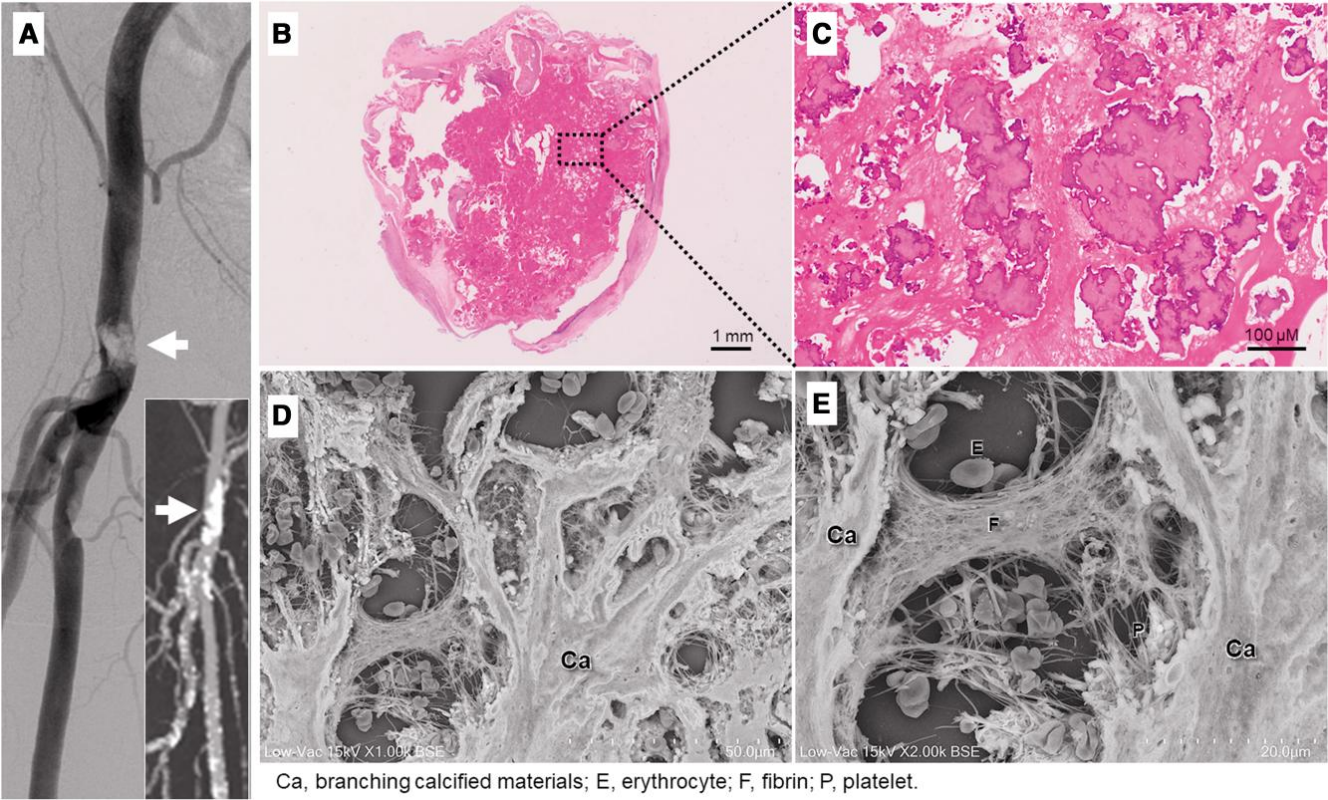


Histopathological analysis of the endarterectomy specimens showed calcified nodules (CNs), which consisted of numerous irregularly shaped micronodules of calcification with interspersed and overlying thrombus (*Panels B* and *C*). Low-vacuum scanning electron microscopy revealed that the calcified materials, which appeared to be numerous small nodular fragments in the thrombus on light microscopy, were actually tightly connected and branched (*Panel D*). Furthermore, distinctive networks of fibrin fibres and platelets capturing erythrocytes were observed in the CNs (*Panel E*).

This is the first report of the three-dimensional structure and detailed surface configuration of CNs in the CFA based on the novel modality of low-vacuum scanning electron microscopy. Treatment for peripheral artery disease with CNs remains challenging. Our findings could contribute to a better understanding of the pathophysiology of CNs.

## Supplementary Material

ytae196_Supplementary_Data

## Data Availability

The data underlying this article are available in the article and in its online Supplementary material.

## Supplementary material

**Consent:** The authors confirm that written consent for submission and publication of this case report, including images and associated text, has been obtained from the patient in line with COPE guidance.

**Funding:** None declared.

